# Prognostic impact of the lipid metabolism gene AGPAT4 in the tumor immune microenvironment of thyroid cancer

**DOI:** 10.1186/s44342-025-00065-0

**Published:** 2026-01-10

**Authors:** Ying Zhu, Wenbo Xu, Xuejing Bai, Yanyuan Qiao, Dan Ye

**Affiliations:** The Sixth Afffiliated Hospital of Kunming Medical University, Yuxi City, Yunnan Province China

**Keywords:** AGPAT4, Thyroid cancer, Prognostic prediction, Immune infiltration, Molecular pathway

## Abstract

**Background:**

Thyroid cancer (THCA) is a common malignant tumor of the endocrine system, and significant clinical challenges remain in its diagnosis and prognostic evaluation. This study aims to elucidate the role of AGPAT4 in thyroid cancer by investigating its expression, involvement in metabolic pathways, and potential as a prognostic biomarker.

**Methods:**

We analyzed data from 512 thyroid cancer patients and 279 controls, performed differential expression analysis of AGPAT4 in thyroid cancer, analyzed the gene expression correlation of AGPAT4 in thyroid cancer, and the protein–protein interaction (PPI) network and functional enrichment analysis of AGPAT4 and its differentially expressed genes (DEGs) were constructed. The Kruskal–Wallis test and receiver operating characteristic (ROC) curve analysis were used to investigate the correlation between AGPAT4 expression and clinicopathological characteristics as well as its diagnostic efficacy. Cox regression analysis and Kaplan–Meier analysis were employed to evaluate its prognostic value. Additionally, single-sample gene set enrichment analysis (ssGSEA) was utilized to explore the association between AGPAT4 expression and the level of immune infiltration in the tumor microenvironment.

**Results:**

Our findings revealed that AGPAT4 was significantly downregulated in thyroid cancer (THCA) tissues (*P* < 0.001), suggesting a potential tumor-suppressive role of AGPAT4 in thyroid cancer. AGPAT4 exhibited robust efficacy in distinguishing tumor tissues from normal tissues, with an area under the receiver operating characteristic curve (AUC) of 0.973. Furthermore, AGPAT4 expression levels were significantly correlated with pathological stage and survival rate (*P* < 0.05). Kaplan–Meier survival analysis showed that patients with high AGPAT4 expression had better progression-free interval (PFI) (HR = 0.45, *P* = 0.007). Protein–protein interaction (PPI) network and functional enrichment analyses revealed that AGPAT4 is involved in key pathways associated with thyroid cancer progression. Immune infiltration analysis suggested an association between AGPAT4 expression and immune responses in the tumor microenvironment.

**Conclusion:**

AGPAT4 holds promise as a potential biomarker for the differential diagnosis and prognostic assessment of thyroid cancer, thereby providing a possible reference for the further exploration of therapeutic strategies against this disease.

**Supplementary Information:**

The online version contains supplementary material available at 10.1186/s44342-025-00065-0.

## Introduction

Thyroid cancer (THCA) is a significant public health concern and one of the most common malignant tumors of the endocrine system, with a globally increasing incidence [1–3]. The pathogenesis of thyroid cancer is multifactorial, involving genetic variations, environmental factors, and abnormal metabolic processes [4]. AGPAT4 (1-acylglycerol-3-phosphate O-acyltransferase 4) is a key enzyme belonging to the lysophosphatidic acid acyltransferase (LPAAT) family. It primarily catalyzes the conversion of lysophosphatidic acid (LPA) to phosphatidic acid (PA) and plays multiple roles in lipid metabolism, particularly in phospholipid synthesis and the regulation of cellular signal transduction [5]. A metabolomic study by Tu et al. demonstrated enhanced lipid metabolic activity in THCA; multi-omic analyses identified six key lipid metabolism genes (LMGs) associated with fatty acid and glycerophospholipid metabolism, namely FABP4, PPARGC1A, AGPAT4, ALDH1A1, TGFA, and GPAT3. This study confirmed that overall survival (OS) was significantly poorer in the high-risk group of THCA patients based on the LMG model, highlighting the potential utility of lipid metabolism-related risk models in guiding clinical treatment and improving prognosis for THCA patients [6]. In recent years, accumulating evidence has indicated that AGPAT4, as a critical lipid metabolic enzyme, is implicated in cancer progression [7–9]. Therefore, investigating the expression and mechanism of AGPAT4 in thyroid cancer will help elucidate its potential role in tumorigenesis and progression, and provide novel insights for clinical diagnosis and therapy.

This study aims to explore the expression and functional implications of AGPAT4 in thyroid cancer (THCA). We conducted bioinformatics analyses using data from The Cancer Genome Atlas (TCGA) and the Gene Expression Omnibus (GEO) database to investigate the differential expression of AGPAT4 between thyroid cancer tissues and normal thyroid tissues. The primary objective of this research is to delineate the expression pattern of AGPAT4, explore its correlation with clinicopathological features, and assess its prognostic significance in thyroid cancer patients. Additionally, a protein–protein interaction (PPI) network was constructed to identify potential downstream genes associated with AGPAT4, followed by functional enrichment analysis to explore the biological pathways involved in these genes.

In conclusion, this study integrated bioinformatic approaches with clinical data analysis to investigate the role of AGPAT4 in thyroid carcinoma, providing evidence to elucidate its molecular mechanisms and suggesting the potential value of AGPAT4 as a diagnostic and prognostic biomarker.

## Methods

### RNA-seq data and bioinformatics analysis

TPM-normalized RNA-seq data of thyroid adenocarcinoma (THCA) were downloaded from the TCGA portal, and corresponding normal thyroid tissue data were obtained from the GTEx portal. Acquisition of expression data: TPM-formatted RNA-seq data from TCGA and GTEx, uniformly processed through the Toil pipeline (Vivian J et al., 2017), were retrieved from UCSC XENA (https://xenabrowser.net/datapages/). THCA-related data from TCGA and corresponding normal tissue data from GTEx were extracted. Data processing methods: log2(value + 1) transformation was performed. Software and R packages: R (version 4.2.1) with R packages including ggplot2 [3.4.4], stats [4.2.1], and car [3.1–0]. Statistical method: Wilcoxon rank sum test. Processing procedure: appropriate statistical methods (from the stats and car packages) were selected based on the characteristics of the data format; statistical analysis was not conducted if the data failed to meet the statistical requirements. Data visualization was performed using the ggplot2 package. This study was conducted in accordance with the Declaration of Helsinki (revised in 2013) and strictly adhered to the publication guidelines of TCGA. No human or animal subjects were involved in this study [10].

### Receiver operating characteristic (ROC) curve analysis

TCGA data corresponding to thyroid cancer and normal tissue data from GTEx were extracted. Data processing and visualization were performed using R software (version 4.2.1). ROC analysis was conducted on the preprocessed data using the R packages “Proc” (version 1.18.0) and “ggplot2” (version 3.4.4), and the results were visualized with ggplot2.

### Clinical statistical analysis, model construction, and prognostic evaluation

To investigate differences in clinicopathological characteristics between groups stratified by AGPAT4 expression levels, data were processed using the log₂(value + 1) transformation with R software (version 4.2.1) and the R package “stats”. For Cox regression analysis: Patients were grouped based on the median AGPAT4 expression value. The R packages “survival” (version 3.3.1) and “rms” (version 6.3–0) were employed. The proportional hazards assumption was tested, and Cox regression analysis was performed using the “survival” package. Kaplan–Meier analysis was conducted with the R packages “survival” (version 3.3.1), “survminer” (version 0.4.9), and “ggplot2” (version 3.4.4). The proportional hazards assumption was verified, and survival regression models were fitted using the “survival” package, with results visualized via the “survminer” and “ggplot2” packages. Based on the results of multivariate Cox regression analysis, a nomogram was constructed by integrating independent prognostic indicators to predict the 1-year, 3-year, and 5-year survival rates of patients. The predictive accuracy of the nomogram was validated using calibration plots to assess its prognostic performance.

### Functional enrichment analysis

Gene sets most strongly correlated with the AGPAT4 gene were selected for Gene Ontology (GO) and Kyoto Encyclopedia of Genes and Genomes (KEGG) enrichment analyses. R software (version 4.2.1) and the clusterProfiler package (version 4.4.4) were used. Processing procedure: after performing ID conversion on the input molecular list using the org.Hs.eg.db package (a dedicated ID conversion tool for human genes), enrichment analysis was conducted with the clusterProfiler package, with the species specified as Homo sapiens. Visualization plots of the enrichment analysis results were generated via the ggplot2 package. Datasets for Gene Set Enrichment Analysis (GSEA) were mainly retrieved from the MSigDB database (https://www.gsea-msigdb.org/gsea/msigdb/index.jsp). The significance thresholds for GSEA results were set as follows: adjusted *P*-value (*p*.adj) < 0.05 and *q*-value < 0.25, with the Benjamini–Hochberg (BH) method used for *P*-value adjustment. Finally, R software (version 4.2.1) and the ggplot2 package (version 3.4.4) were employed to visualize the GSEA enrichment results as ridge plots.

## Cell culture and detection

### Cell culture and transfection

The NTHY-ORI3 cell line was cultured in RPMI1640 medium. The TPC-1 and 8505 C cell lines were cultured in DMEM medium (GIBCO BRL, Grand Island, NY, USA) supplemented with 10% fetal bovine serum (FBS), 100 IU/mL penicillin, and 100 μg/mL streptomycin. All cells were incubated in a humidified incubator at 37 °C with 5% CO₂.

### RNA extraction and quantitative real-time reverse transcription PCR (qRT-PCR)

Total RNA was extracted from cells using the mirVana miRNA Isolation Kit (Ambion, Austin, TX, USA) according to the manufacturer’s protocol. Quantitative real-time reverse transcription PCR (qRT-PCR) was performed to detect the relative transcriptional level of AGPAT4. The PCR reaction conditions were as follows: initial denaturation at 94 °C for 4 min; followed by 40 cycles of denaturation at 94 °C for 30 s, annealing at 56 °C for 30 s, and extension at 72 °C for 30 s. The relative expression level of the target gene was calculated using the 2ΔΔCt method. All primers were synthesized by AuGCT Inc. (Beijing, China) (Supplementary Table S1).

### Western blotting

Cultured cells were lysed in RIPA lysis buffer, and the cell lysates were analyzed by standard Western blotting procedures. Glyceraldehyde-3-phosphate dehydrogenase (GAPDH) was used as an endogenous reference protein. The specific antibodies used were purchased from Saierbio (Tianjin, China).

### Statistical analysis

Statistical analyses were performed using R (version 4.2.1). Appropriate statistical methods were selected for intergroup difference analysis based on data types: the Wilcoxon rank sum test was used for unpaired samples, the Wilcoxon signed rank test for paired samples, and the two-sided Student’s *t*-test for parametric data. Associations between continuous variables were evaluated via Spearman’s correlation analysis. To address the issue of multiple comparisons across multiple genes or pathways, *P*-values were adjusted using the Benjamini–Hochberg false discovery rate (FDR) method. A *P*-value < 0.05 was considered statistically significant [11, 12].

## Results

### Expression of AGPAT4 in thyroid cancer (THCA)

**Fig. 1:**
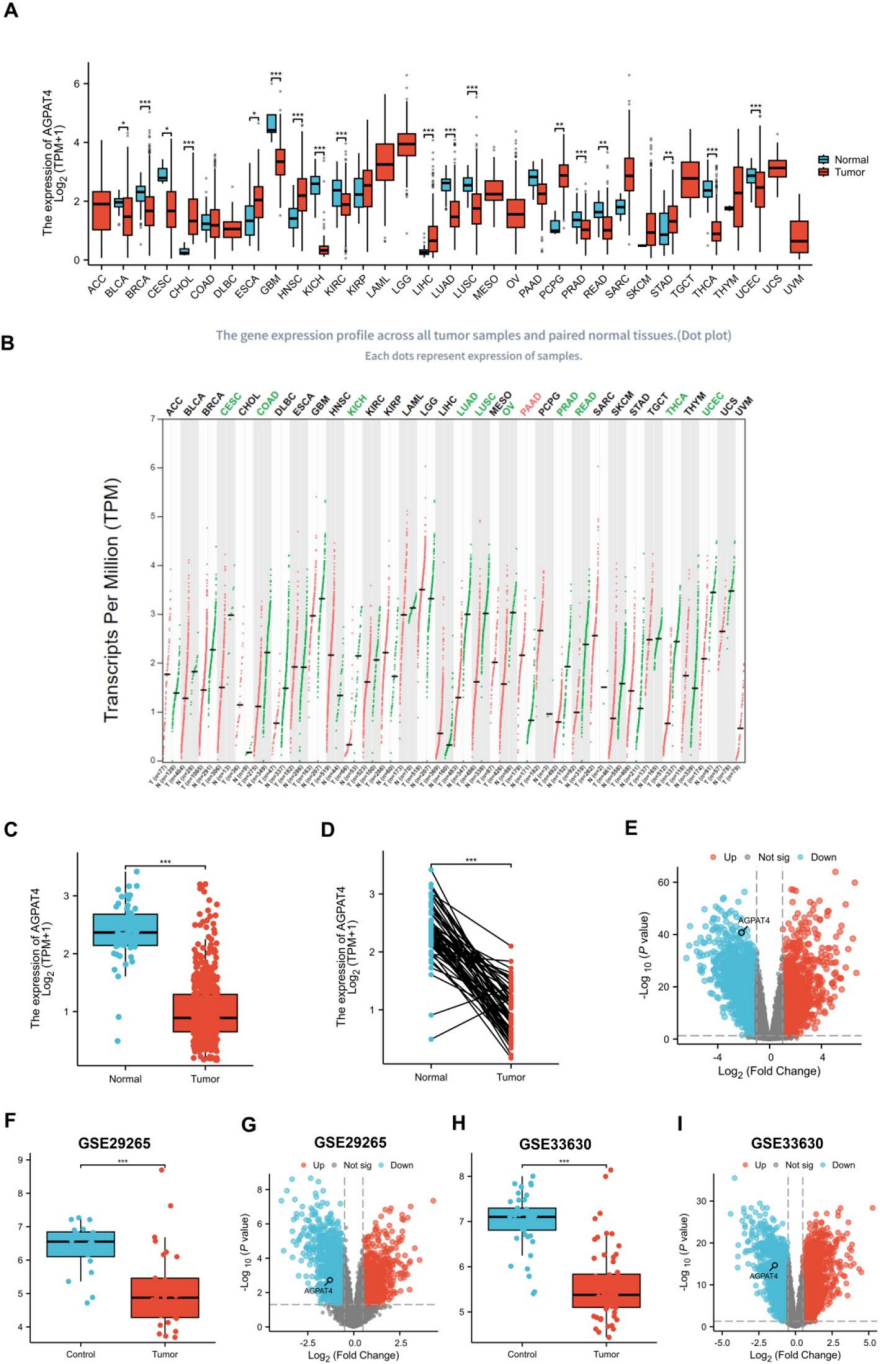
Differential expression of AGPAT4 in pan-cancer and thyroid cancer tumor tissues. **A** and **B** show the expression of AGPAT4 in unpaired and paired samples, respectively, from the TCGA database and GEPIA2. **C** and **D** present the expression of AGPAT4 in unpaired and paired samples, respectively, from the TCGA–THCA database. **E** Volcano plot of differentially expressed genes in thyroid cancer. **F**–**I **The GSE33630 and GSE29265 datasets were utilized to verify AGPAT4 gene expression in thyroid cancer. *P < 0.05; **P < 0.01; ***P < 0.001

**Fig. 2:**
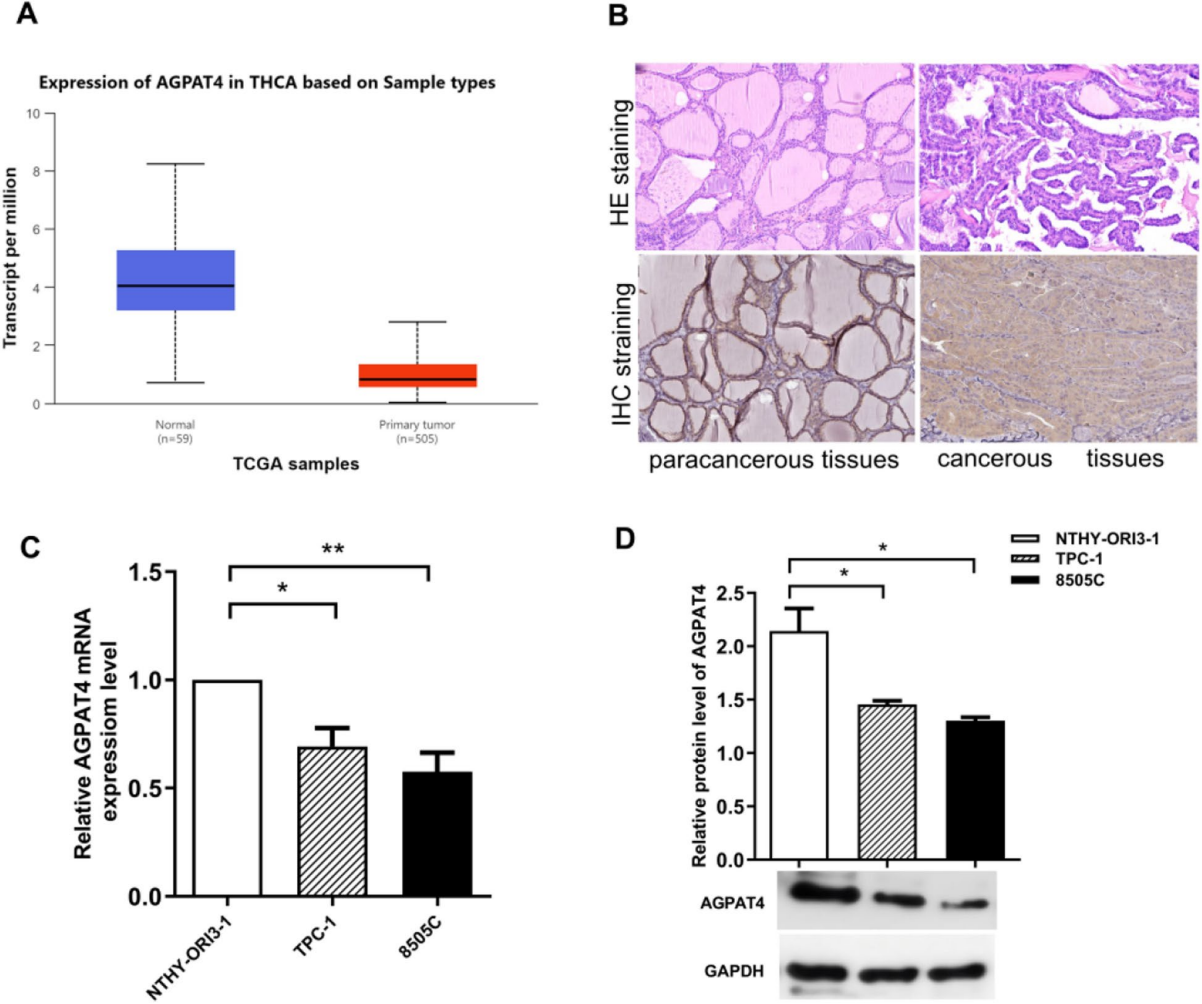
Protein expression of AGPAT4 in thyroid cancer. **A** Analysis via the CPTAC (Clinical Proteomic Tumor Analysis Consortium) database showed that the protein expression level of AGPAT4 in thyroid cancer tissues was lower than that in adjacent non-cancerous tissues. **B** Representative images of histopathological sections stained with hematoxylin and eosin (HE) and immunohistochemical (IHC) staining of AGPAT4 in adjacent non-cancerous tissues and cancerous tissues of thyroid cancer. Representative images were obtained from the Human Protein Atlas. **C** The mRNA expression levels of AGPAT4 detected in three cell types (NTHY-ORI3, TPC-1, and 8505 C). **D** The protein expression levels of AGPAT4 detected in the same three cell types

First, we performed a differential expression analysis of AGPAT4 across multiple cancer types and found that it was differentially expressed in 18 tumor types, including thyroid cancer (THCA) (*P* < 0.001) (Fig. 1A). In pan-cancer paired samples, AGPAT4 expression was significantly downregulated in thyroid cancer and various other malignant tumor tissues (Fig. 1B). Analysis results of paired and unpaired samples from the TCGA-THCA database (Fig. 1C, D) showed that AGPAT4 expression in tumor tissues was significantly lower than that in normal tissues (*P* < 0.001). Furthermore, we validated the aforementioned expression differences using thyroid cancer transcriptome data: Fig. 1E shows a volcano plot of the thyroid cancer gene expression profile. Meanwhile, the GSE33630 and GSE29265 datasets from the GEO database were selected for validation. The results showed that the expression level of AGPAT4 in tumor tissues was significantly lower than that in normal tissues (*P* < 0.001), with a significant downregulation in thyroid cancer tissues. Analysis of the CPTAC database showed that the protein abundance of AGPAT4 in thyroid cancer tissues was lower than that in normal tissues (Fig. 2A). Figure 2B presents hematoxylin–eosin (HE)-stained pathological sections of normal tissues adjacent to thyroid cancer and tumor tissues, as well as immunohistochemical (IHC) staining sections for AGPAT4. The results revealed that the staining intensity of the AGPAT4 protein in thyroid cancer tissues was significantly weaker compared to adjacent normal tissues.

In addition, we detected AGPAT4 expression in three cell lines (NTHY-ORI3, TPC-1, 8505 C) (Fig. 2C, D). Compared with normal thyroid cells, both mRNA and protein levels of AGPAT4 were significantly decreased in thyroid cancer cells. Collectively, these experimental findings indicate that AGPAT4 holds potential as a biomarker for thyroid cancer.

### Analysis of gene expression correlation of AGPAT4 in thyroid cancer (THCA)

**Fig. 3:**
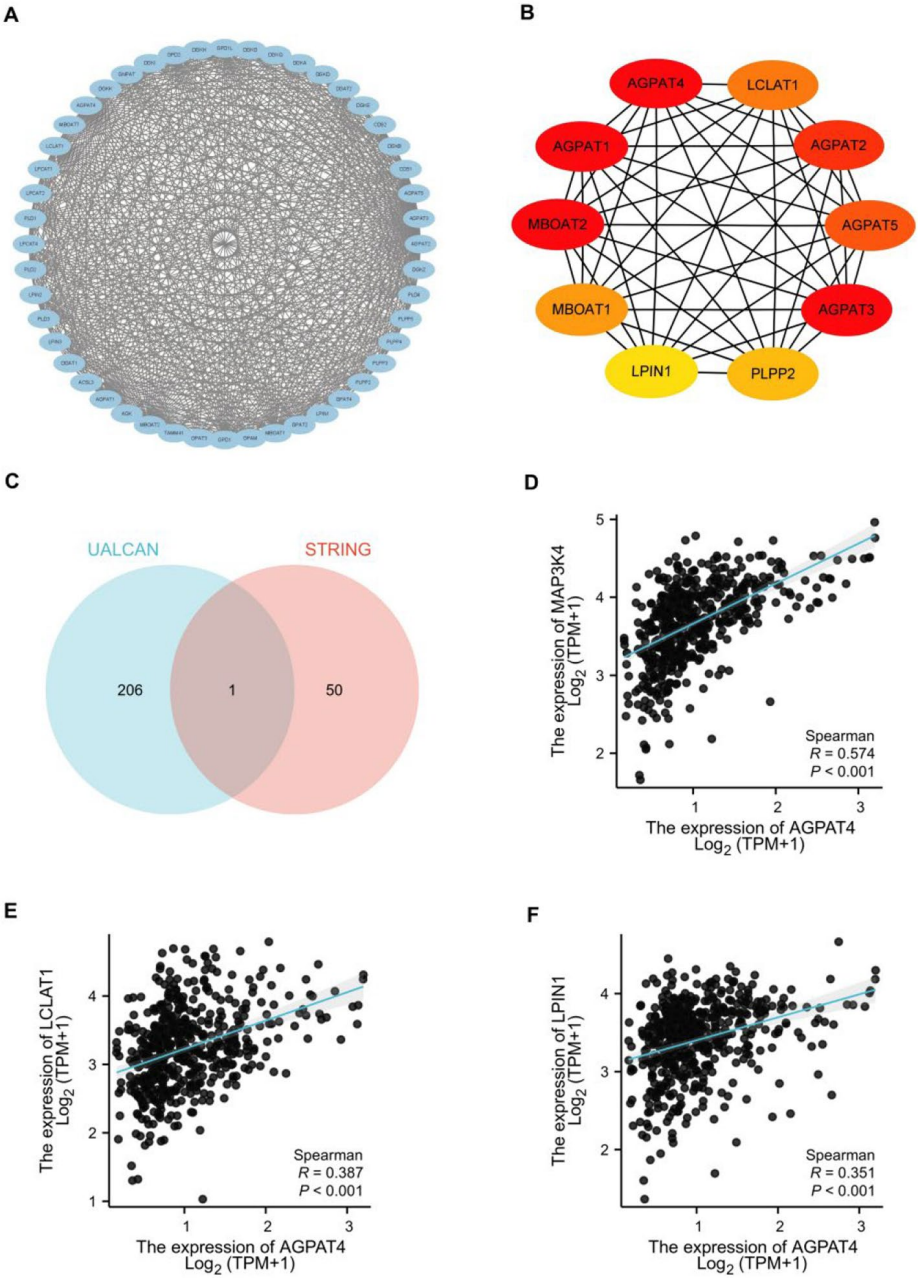
Gene correlation analysis of AGPAT4 in thyroid cancer. **A** Protein–protein interaction (PPI) network. **B** PPI network of hub genes. **C** Venn diagram of UALCAN and STRING databases in papillary thyroid carcinoma (THCA). **D**–**F** Correlation analysis between AGPAT4 and three genes—MAP3K4, LCLAT1, and LPIN1

Fifty genes related to AGPAT4 were screened from the STRING database, and a specific protein–protein interaction (PPI) network was constructed using Cytoscape software (Fig. 3A), with nine hub genes identified, including AGPAT1, AGPAT2, MBOAT1, MBOAT2, AGPAT3, AGPAT5, LCLAT1, PLPP2, and LPIN1 (Fig. 3B). Subsequently, a Venn diagram was generated to screen for AGPAT4-related common genes in thyroid cancer (THCA) from the UALCAN database (206 genes) and the STRING database (50 genes) (Fig. 3C), and MAP3K4 was identified as the intersection gene. Based on this, two key lipid metabolism-related genes from the interaction network and one intersection gene (LCLAT1, LPIN1, MAP3K4) were selected for expression correlation analysis with AGPAT4. The results demonstrated that all three genes were positively correlated with AGPAT4 (Fig. 3D, E, F).

### Pathway enrichment analysis of AGPAT4 in thyroid cancer (THCA)

**Fig. 4:**
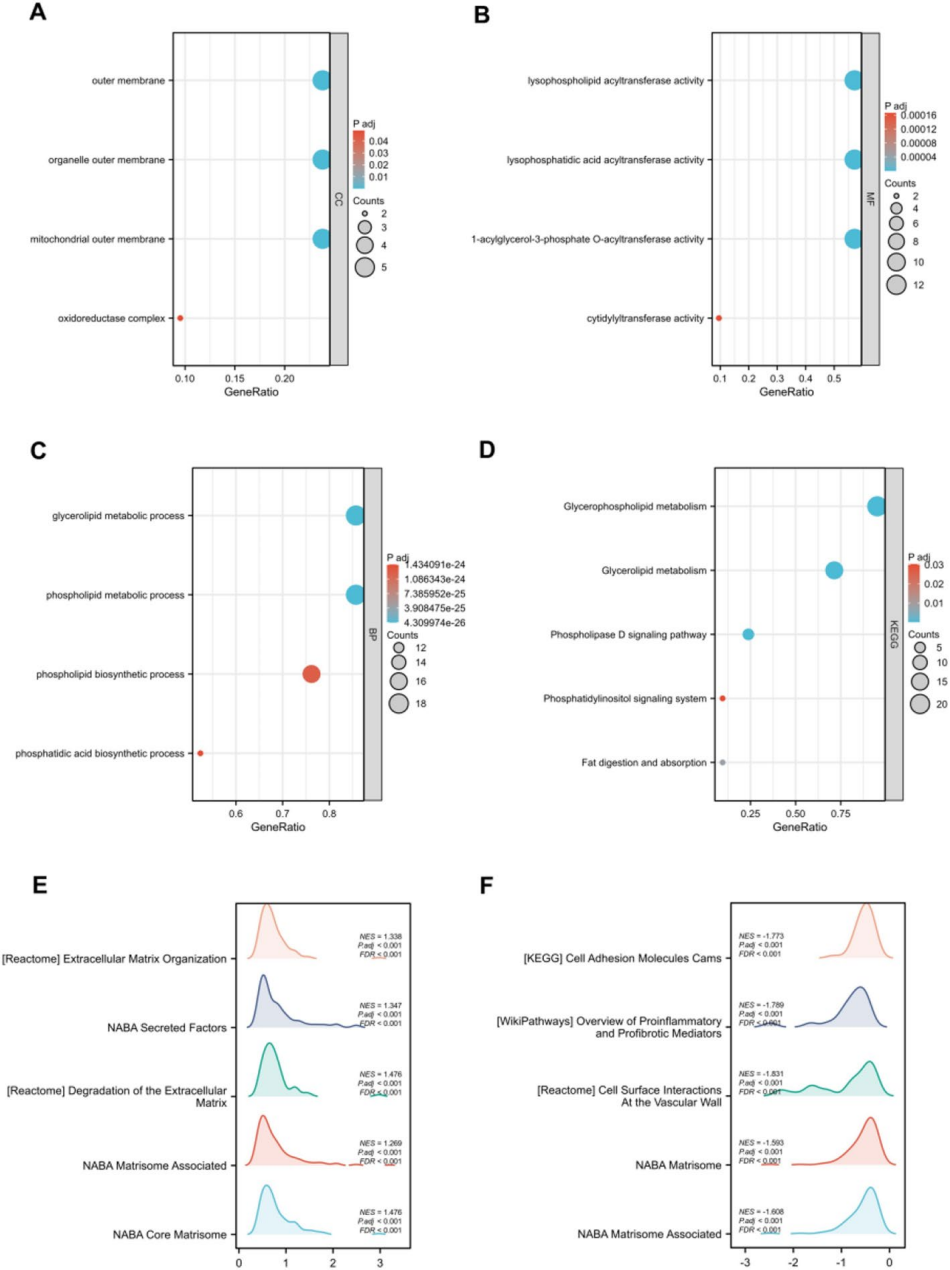
Functional enrichment analysis of AGPAT4-related differentially expressed genes (DEGs) in thyroid cancer. **A**–**D** GO and KEGG enrichment analyses of AGPAT4-related DEGs. **E**, **F** The most significantly enriched pathways between the AGPAT4-low expression group and AGPAT4-high expression group identified by Gene Set Enrichment Analysis (GSEA)

GO enrichment analysis was performed on differentially expressed genes associated with AGPAT4 (Fig. 4A–C). The results showed that these genes are involved in cellular components including the outer membrane and mitochondrial outer membrane; molecular functions including lysophospholipid acyltransferase activity and 1-acylglycerol-3-phosphate O-acyltransferase activity; and biological processes including glycerolipid metabolic process, phospholipid metabolic process, and phospholipid biosynthetic process. Subsequently, KEGG enrichment analysis was conducted (Fig. 4D), revealing that AGPAT4 is associated with pathways such as glycerophospholipid metabolism, glycerolipid metabolism, and phospholipase D signaling pathway. These findings suggest the core role of AGPAT4 in the lipid metabolism network (Supplementary Fig. S1).

Finally, we performed Gene Set Enrichment Analysis (GSEA) and pathway screening on the differentially expressed genes associated with AGPAT4. The results showed that a total of 10 pathways were significantly enriched, including Extracellular Matrix Organization, NABA Secreted Factors, Degradation of the Extracellular Matrix, Cell Adhesion Molecules (Cams), Overview of Proinflammatory and Profibrotic Mediators, and Cell Surface Interactions at the Vascular Wall (Fig. 4E, F).

### Relationship between AGPAT4 expression and immune infiltration

**Fig. 5:**
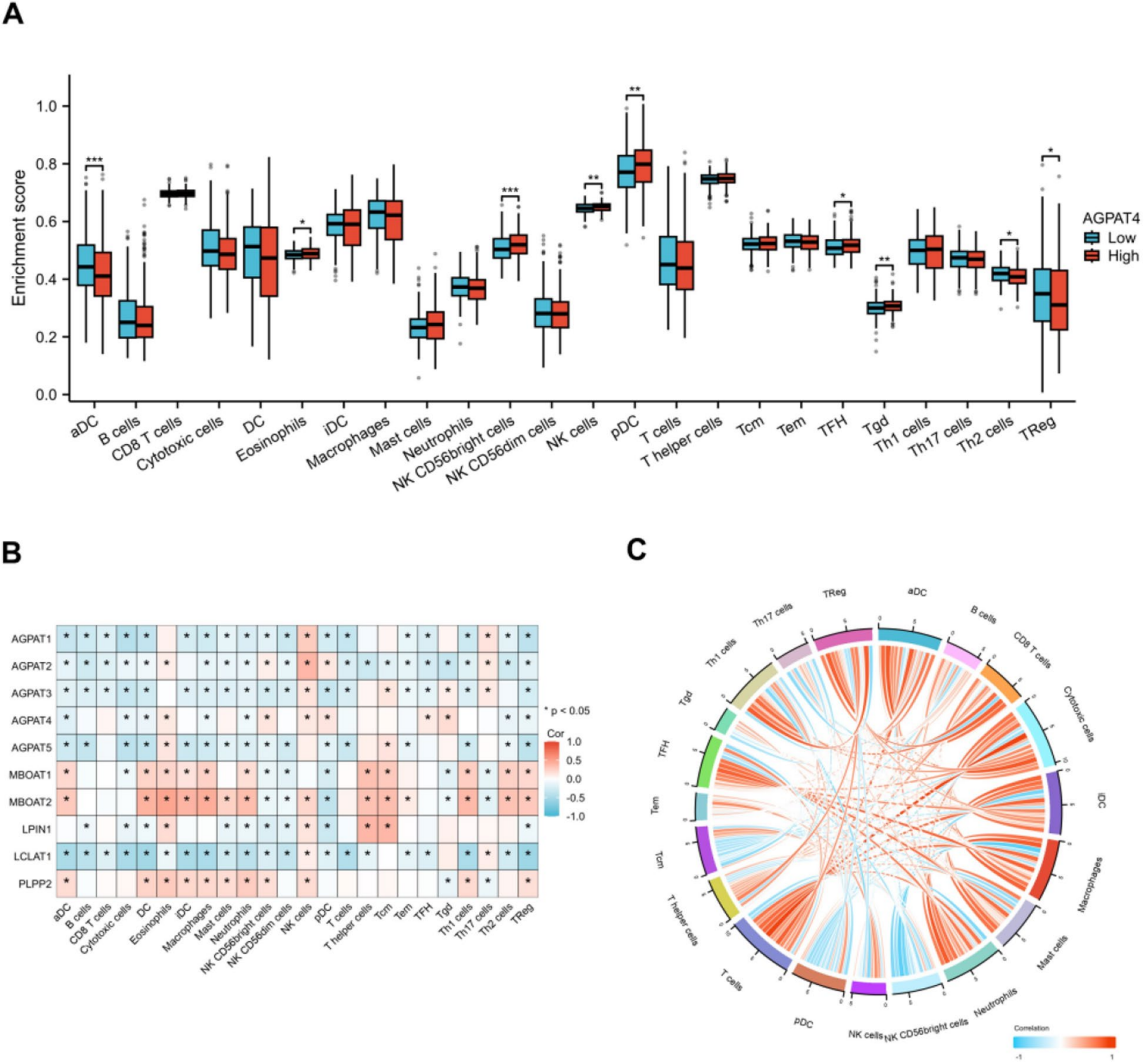
Immune infiltration analysis of AGPAT4. **A** Box plots comparing enrichment scores for 24 immune cell types between the AGPAT4 high-expression and low-expression groups. **B** Heatmap showing the correlation between genes and immune cell infiltration. **C** Chord diagram illustrating the correlations between gene expression and immune cell infiltration, where blue lines indicate negative correlations and red lines indicate positive correlations. *P < 0.05, **P < 0.01, ***P < 0.001

Statistically significant differences in immune infiltration scores were observed between the AGPAT4 high- and low-expression groups for activated dendritic cells (aDC), NK CD56bright cells, NK cells, plasmacytoid dendritic cells (pDC), follicular helper T (TFH) cells, Tgd, Th2 cells, and TReg (*P* < 0.05; Fig. 5A). In addition, the correlation heatmap between gene expression and immune cell infiltration levels (Fig. 5B) illustrates the significance of correlations between the nine hub genes of AGPAT4 and various immune cell subsets: red indicates a positive correlation (the closer the correlation coefficient is to 1, the stronger the positive correlation), while blue indicates a negative correlation (the closer the correlation coefficient is to − 1, the stronger the negative correlation). Furthermore, a correlation chord diagram was constructed based on AGPAT4 expression levels and the infiltration scores of individual immune cells (Fig. 5C). Collectively, these results demonstrate that the immune infiltration status in thyroid cancer is closely associated with AGPAT4 expression levels.

### Relationship between AGPAT4 expression and clinicopathological features

Statistical analysis was performed on the baseline data of 512 thyroid cancer (THCA) patients retrieved from the TCGA database (Table 1). The data were divided into two groups, each containing 256 samples: one group with low AGPAT4 expression and the other with high AGPAT4 expression, based on the median expression level. There were no significant differences between the two groups with respect to gender (*P* = 0.487), pathological stage (*P* = 0.558), lymph node (N) stage (*P* = 0.114), or metastasis (M) stage (*P* = 0.779). However, significant differences were observed in the distribution of T stages (*P* = 0.045), extrathyroidal extension (*P* = 0.023), overall survival (*P* = 0.042), and progression-free interval (PFI) events (*P* = 0.004).

**Table 1:** The relationship between AGPAT4 gene expression and clinicopathological factors

Characteristics	Low expression of AGPAT4	High expression of AGPAT4	*P* value
*n*	256	256	
Pathologic T stage, *n *(%)			**0.045**
T1	60 (11.8%)	83 (16.3%)	
T2	86 (16.9%)	83 (16.3%)	
T3&T4	110 (21.6%)	88 (17.3%)	
Pathologic N stage, *n* (%)			0.114
N0	106 (22.9%)	123 (26.6%)	
N1	125 (27.1%)	108 (23.4%)	
Pathologic M stage, *n* (%)			0.779
M0	129 (43.7%)	157 (53.2%)	
M1	5 (1.7%)	4 (1.4%)	
Pathologic stage, *n* (%)			0.558
Stage I	136 (26.7%)	152 (29.8%)	
Stage II	28 (5.5%)	24 (4.7%)	
Stage III	60 (11.8%)	53 (10.4%)	
Stage IV	31 (6.1%)	26 (5.1%)	
Gender, *n* (%)			0.487
Female	190 (37.1%)	183 (35.7%)	
Male	66 (12.9%)	73 (14.3%)	
Extrathyroidal extension, *n *(%)			**0.023**
No	159 (32.2%)	181 (36.6%)	
Yes	89 (18%)	65 (13.2%)	
OS event, *n *(%)			**0.042**
Alive	252 (49.2%)	244 (47.7%)	
Dead	4 (0.8%)	12 (2.3%)	
PFI event, *n *(%)			**0.004**
No	219 (42.8%)	239 (46.7%)	
Yes	37 (7.2%)	17(3.3%)	

### Suggestive significance of AGPAT4 for prognosis in patients with thyroid cancer (THCA)

**Fig. 6:**
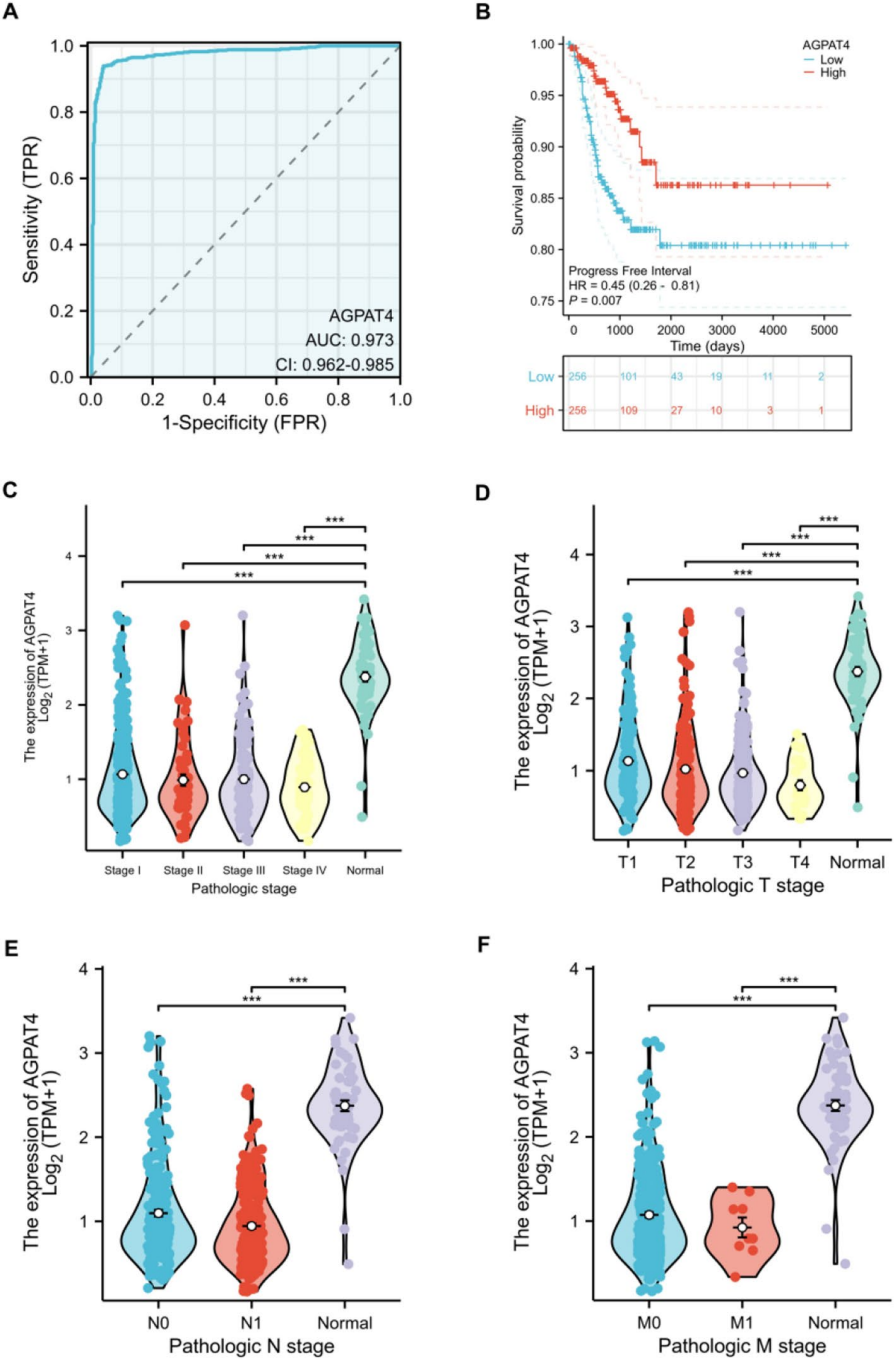
Correlation analysis between AGPAT4 gene expression and clinicopathological factors. **A** Receiver Operating Characteristic (ROC) curve: Demonstrates the efficacy of AGPAT4 in distinguishing thyroid cancer tissues from normal tissues. **B** Kaplan–Meier (K-M) survival analysis: Comparison of Progression-Free Interval (PFI) between the AGPAT4 low-expression and high-expression groups in thyroid cancer. **C** Expression differences of AGPAT4 across different pathological stages. **D** Expression differences of AGPAT4 across different T stages. **E** Expression differences of AGPAT4 across different N stages. **F** Expression differences of AGPAT4 across different M stages; *P < 0.05, **P < 0.01, ***P < 0.001

We used a ROC curve to analyze the diagnostic efficacy of AGPAT4 in differentiating tumor tissues from non-tumor tissues. The area under the curve (AUC) for AGPAT4 was 0.973 (CI = 0.962–0.985), indicating high diagnostic accuracy (Fig. 6A). Kaplan–Meier survival analysis evaluated the prognostic value of AGPAT4 expression in thyroid cancer. The progression-free interval (PFI) survival curve demonstrated that patients with high AGPAT4 expression had a higher survival rate than those with low AGPAT4 expression, and this difference was statistically significant (HR = 0.45[0.26–0.81], *P* = 0.007) (Fig. 6B). Furthermore, Fig. 6C–F demonstrates that the correlation between AGPAT4 expression and clinical parameters varies significantly. Regarding pathological stages, AGPAT4 expression in normal tissue samples was significantly higher than in patients with stage I, stage II, stage III, and stage IV disease (*P* < 0.001). Similarly, in TNM staging, AGPAT4 expression in normal tissue samples was significantly higher than in patients with M0 stage, M1 stage, N0 stage, N1 stage, T1 stage, T2 stage, T3 stage, and T4 stage disease (*P* < 0.001).

### Construction and validation of the AGPAT4 prognostic nomogram

**Fig. 7:**
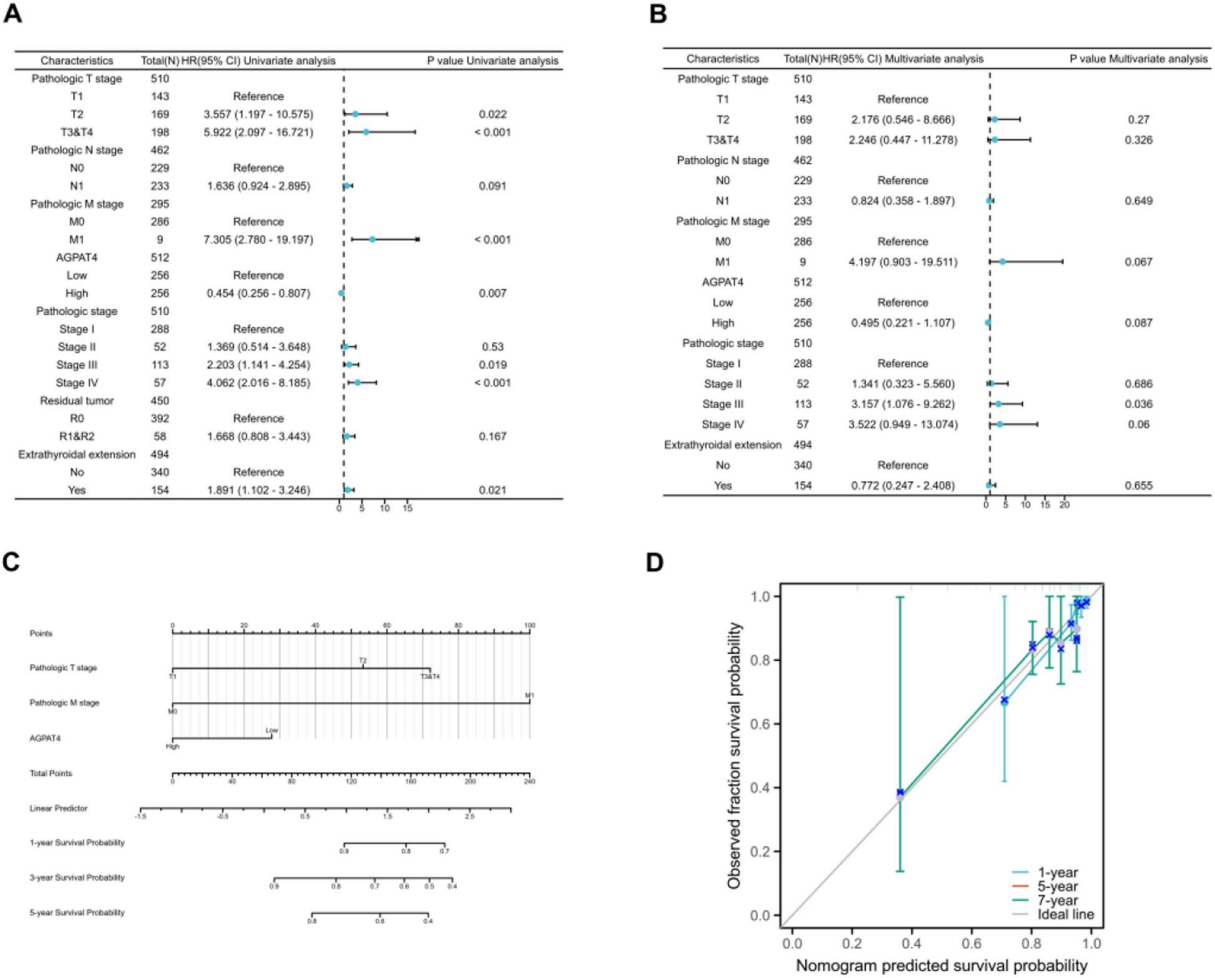
Construction and Validation of the Prognostic Nomogram Model. **A** Forest plot of univariate Cox analysis based on progression-free interval (PFI). **D** Forest plot of multivariate Cox analysis based on progression-free interval (PFI). **B** Nomogram for predicting 1-year, 3-year, and 5-year progression-free interval (PFI) of thyroid cancer (THCA) patients. **C** Calibration curves of the nomogram for predicting 1-year, 5-year, and 7-year progression-free interval (PFI)

The results of univariate survival analysis showed that T stage, M stage, AGPAT4 expression level, pathological stage (Stage III/IV), and extrathyroidal extension were significantly associated with patient prognosis (*P* < 0.05); in contrast, N stage and residual tumor were not significantly correlated with prognosis (Fig. 7A). The results of multivariate survival analysis indicated that AGPAT4 expression did not exhibit a significant independent association with patient prognosis (Fig. 7B).

In addition, a prognostic nomogram for thyroid cancer (THCA) patients was constructed by integrating TNM stage and AGPAT4 expression level to predict patient prognosis. The concordance index (C-index) of this nomogram was 0.71 (95% confidence interval 0.68–0.74), indicating moderate predictive accuracy of the model (Fig. 7C). Subsequently, a calibration curve was plotted (Fig. 7D) to evaluate the predictive precision of the model. The bias-corrected calibration curve was close to the ideal 45° line, demonstrating good consistency between the predicted values of the model and the actual outcomes.

## Discussion

Thyroid cancer is the most common malignant tumor in the endocrine system, with its incidence increasing significantly over the past few decades [13, 14]. Although most thyroid cancers have a favorable prognosis, some patients develop highly aggressive subtypes with poor outcomes, posing substantial challenges to clinical treatment [15–17]. AGPAT4 (1-acylglycerol-3-phosphate O-acyltransferase 4) is a key enzyme belonging to the lysophosphatidic acid acyltransferase (LPAAT) family, primarily responsible for converting lysophosphatidic acid (LPA) to phosphatidic acid (PA) [18]. Its unique molecular structure endows it with a critical role in lipid metabolism, and its functions in regulating cellular signal transduction and metabolism make it a potential biomarker and therapeutic target [19–21]. We constructed a protein–protein interaction (PPI) network centered on AGPAT4 using the STRING database, confirming that AGPAT4 interacts directly with multiple well-characterized lipid metabolic enzymes (e.g., AGPAT3, PLPP4, MBOAT1). Kyoto Encyclopedia of Genes and Genomes (KEGG) pathway enrichment analysis revealed significant enrichment of AGPAT4 in pathways such as “Glycerophospholipid metabolism” and “Glycerolipid metabolism” (FDR < 0.001), strongly supporting its important role in the lipid metabolic network at the systemic level.

Previous studies have demonstrated that silencing AGPAT4 inhibits tumor cell growth and promotes anti-tumor immune responses by regulating macrophage polarization in colorectal cancer [22]. In contrast, our findings show that AGPAT4 is significantly downregulated in thyroid cancer tissues (*P* < 0.001), suggesting a tumor-suppressive role during thyroid carcinogenesis. AGPAT4 exhibits excellent discriminative power between tumor and normal tissues, with an area under the receiver operating characteristic curve (AUC) of 0.973. Additionally, AGPAT4 expression levels are significantly correlated with pathological stage and survival rate (*P* < 0.05), indicating that low AGPAT4 expression may be associated with thyroid cancer progression. Kaplan–Meier survival analysis for progression-free interval (PFI) showed that patients with high AGPAT4 expression had better survival outcomes than those with low expression (HR = 0.45, *P* = 0.007). Furthermore, AGPAT4 expression in normal tissue samples was significantly higher than that in other groups across pathological stages and TNM stages (*P* < 0.001). Cox analysis revealed that low AGPAT4 expression is a risk factor for poor prognosis (*P* < 0.05), suggesting its potential as a prognostic biomarker for thyroid cancer (THCA). Immune infiltration analysis also indicated an association between AGPAT4 expression levels and the host’s immune response to thyroid cancer in the tumor microenvironment.

Our results suggest that AGPAT4 has the potential to serve as a biomarker for thyroid cancer, particularly in distinguishing tumor tissues from normal tissues and assessing patient prognosis. However, this study still has the following limitations. Firstly, it relies on retrospective data from The Cancer Genome Atlas (TCGA) database, which may introduce inherent biases such as selection bias and heterogeneity in clinical management across different institutions. Secondly, conflicting results were observed between Kaplan–Meier curves for overall survival (OS) and progression-free interval (PFI): our analysis showed that high AGPAT4 expression was associated with shorter OS but longer PFI (Supplementary Fig. S2). We further analyzed the correlation between the expression level of this gene and the clinical stage of thyroid cancer, and the results showed that AGPAT4 expression was higher in T1/T2 than in T3/T4 in pathological T stage, higher in M0 than in M1 in pathological M stage, and higher in N0 than in N1 in pathological N stage; AGPAT4 expression was significantly higher in cases without extrathyroidal extension than in those with extrathyroidal extension (Supplementary Fig. S2). Therefore, this contradiction suggests that AGPAT4 may not directly affect rapid tumor progression (hence the longer PFI), but could ultimately impact OS by increasing patients’ sensitivity to treatment toxicity, inducing fatal complications (e.g., thrombosis, immune-related adverse events), or elevating the risk of non-cancer-related death. Thus, the functional significance of AGPAT4 in thyroid cancer progression requires further investigation through in vitro and in vivo experiments to elucidate its underlying mechanisms.

In conclusion, AGPAT4 exhibits a downregulated expression trend in thyroid cancer, and its expression level is associated with the clinical characteristics of tumors and patient prognosis, thus suggesting that AGPAT4 has potential value as a prognostic biomarker. In addition, the metabolic pathways involving AGPAT4 are linked to immune responses in the tumor microenvironment, which provides a reference direction for further exploring therapeutic strategies for thyroid cancer. This study conducts an in-depth analysis and supplementation of the research by Tu et al. In future research, we will focus on investigating the potential mechanisms by which AGPAT4 regulates specific biological behaviors of tumors such as proliferation and metastasis, and verify its clinical applicability through prospective trials.

## Supplementary Information

Supplementary Material 1. Supplementary Figure S1: (A) Protein–protein interaction (PPI) network; (B-E) GO and KEGG enrichment analyses of AGPAT4-related DEGs; (F) Gene Set Enrichment Analysis (GSEA) of the AGPAT4 gene set. Supplementary Figure S2: (A) Kaplan–Meier (K-M) survival analysis: Comparison of Progression-Free Interval (PFI) between the AGPAT4 low-expression and high-expression groups in thyroid cancer; (B) Kaplan–Meier (K-M) survival analysis: Comparison of overall survival (OS) between the AGPAT4 low-expression and high-expression groups in thyroid cancer; (C-G) Analysis of the correlation between AGPAT4 expression levels and clinicopathological features of thyroid cancer. * *P* < 0.05; **P < 0.01; *** *P *< 0.001. Supplementary Table S1: qRT-PCR Primer Sequences

## Data Availability

No datasets were generated or analysed during the current study.
