# Supplementary figures and images for: Prognostic impact of the lipid metabolism gene AGPAT4 in the tumor immune microenvironment of thyroid cancer
Source: Genomics Inform. 2026 Jan 10;24:1. doi: 10.1186/s44342-025-00065-0 (PMC12879322; doi:10.1186/s44342-025-00065-0)

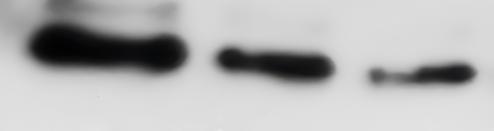

Supplement: Supplementary file 1 — Supplementary Material 1. Supplementary Figure S1: (A) Protein–protein interaction (PPI) network; (B-E) GO and KEGG enrichment analyses of AGPAT4-related DEGs; (F) Gene Set Enrichment Analysis (GSEA) of the AGPAT4 gene set. Supplementary Figure S2: (A) Kaplan–Meier (K-M) survival analysis: Comparison of Progression-Free Interval (PFI) between the AGPAT4 low-expression and high-expression groups in thyroid cancer; (B) Kaplan–Meier (K-M) survival analysis: Comparison of overall survival (OS) between the AGPAT4 low-expression and high-expression groups in thyroid cancer; (C-G) Analysis of the correlation between AGPAT4 expression levels and clinicopathological features of thyroid cancer. * P < 0.05; **P < 0.01; *** P < 0.001. Supplementary Table S1: qRT-PCR Primer Sequences [file 44342_2025_65_MOESM1_ESM.zip › Original Image/AGPAT4.jpg]

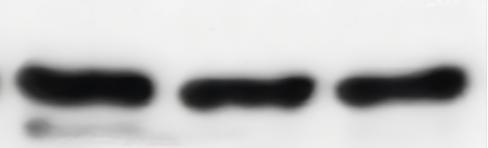

Supplement: Supplementary file 1 — Supplementary Material 1. Supplementary Figure S1: (A) Protein–protein interaction (PPI) network; (B-E) GO and KEGG enrichment analyses of AGPAT4-related DEGs; (F) Gene Set Enrichment Analysis (GSEA) of the AGPAT4 gene set. Supplementary Figure S2: (A) Kaplan–Meier (K-M) survival analysis: Comparison of Progression-Free Interval (PFI) between the AGPAT4 low-expression and high-expression groups in thyroid cancer; (B) Kaplan–Meier (K-M) survival analysis: Comparison of overall survival (OS) between the AGPAT4 low-expression and high-expression groups in thyroid cancer; (C-G) Analysis of the correlation between AGPAT4 expression levels and clinicopathological features of thyroid cancer. * P < 0.05; **P < 0.01; *** P < 0.001. Supplementary Table S1: qRT-PCR Primer Sequences [file 44342_2025_65_MOESM1_ESM.zip › Original Image/GAPDH(1).jpg]

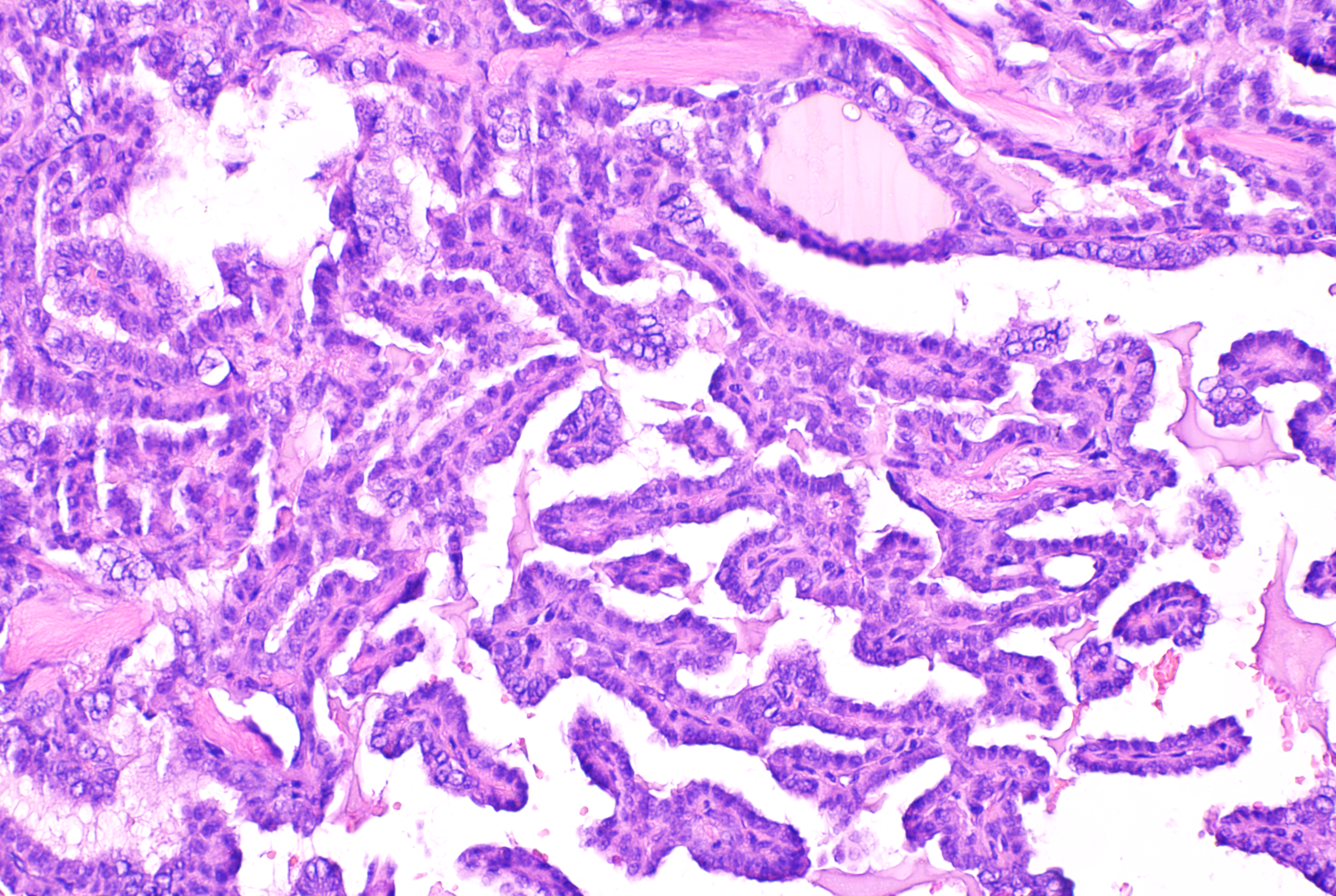

Supplement: Supplementary file 1 — Supplementary Material 1. Supplementary Figure S1: (A) Protein–protein interaction (PPI) network; (B-E) GO and KEGG enrichment analyses of AGPAT4-related DEGs; (F) Gene Set Enrichment Analysis (GSEA) of the AGPAT4 gene set. Supplementary Figure S2: (A) Kaplan–Meier (K-M) survival analysis: Comparison of Progression-Free Interval (PFI) between the AGPAT4 low-expression and high-expression groups in thyroid cancer; (B) Kaplan–Meier (K-M) survival analysis: Comparison of overall survival (OS) between the AGPAT4 low-expression and high-expression groups in thyroid cancer; (C-G) Analysis of the correlation between AGPAT4 expression levels and clinicopathological features of thyroid cancer. * P < 0.05; **P < 0.01; *** P < 0.001. Supplementary Table S1: qRT-PCR Primer Sequences [file 44342_2025_65_MOESM1_ESM.zip › Original Image/╝╫╫┤╧┘╚Θ═╖╫┤░⌐ 20▒╢.tif]

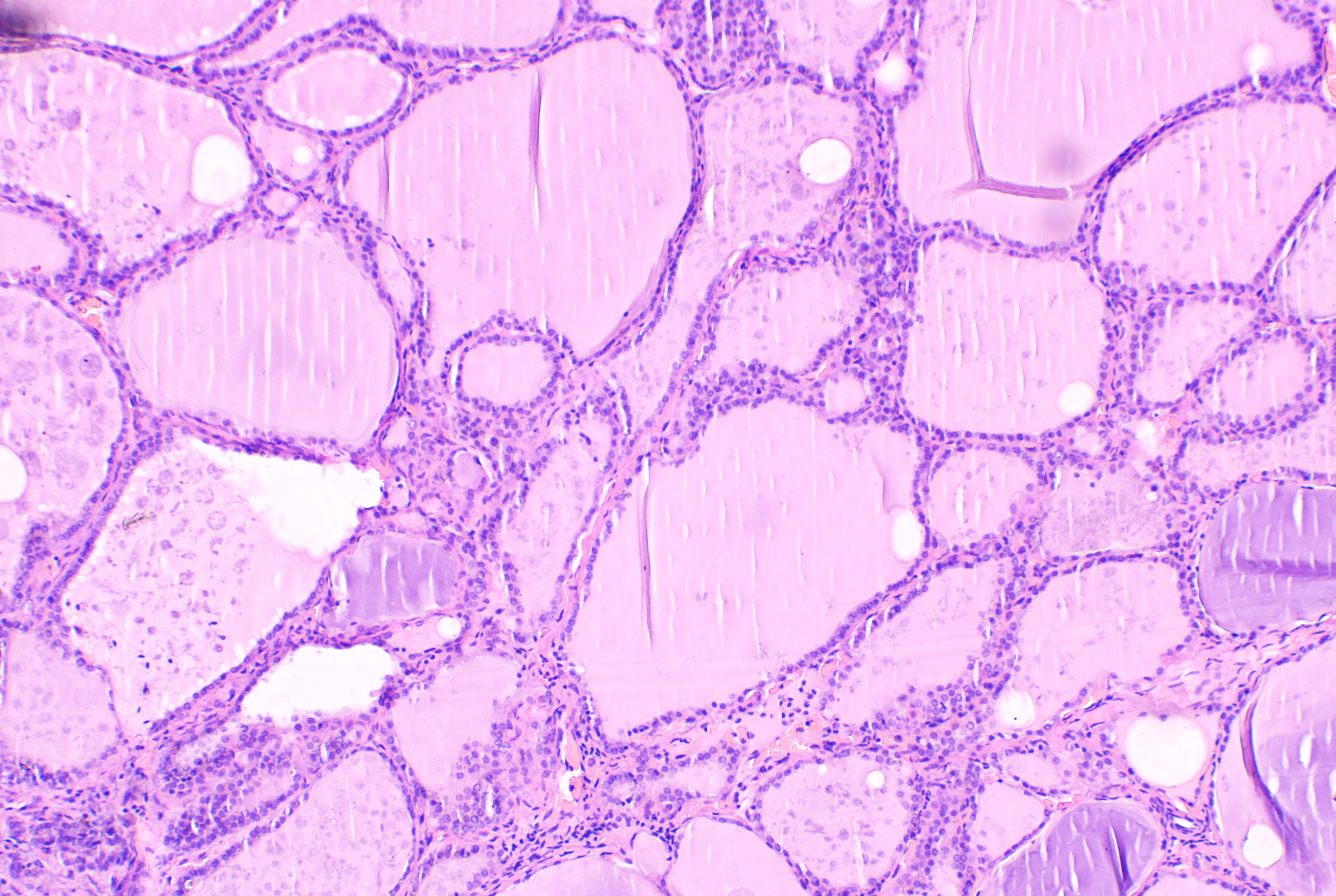

Supplement: Supplementary file 1 — Supplementary Material 1. Supplementary Figure S1: (A) Protein–protein interaction (PPI) network; (B-E) GO and KEGG enrichment analyses of AGPAT4-related DEGs; (F) Gene Set Enrichment Analysis (GSEA) of the AGPAT4 gene set. Supplementary Figure S2: (A) Kaplan–Meier (K-M) survival analysis: Comparison of Progression-Free Interval (PFI) between the AGPAT4 low-expression and high-expression groups in thyroid cancer; (B) Kaplan–Meier (K-M) survival analysis: Comparison of overall survival (OS) between the AGPAT4 low-expression and high-expression groups in thyroid cancer; (C-G) Analysis of the correlation between AGPAT4 expression levels and clinicopathological features of thyroid cancer. * P < 0.05; **P < 0.01; *** P < 0.001. Supplementary Table S1: qRT-PCR Primer Sequences [file 44342_2025_65_MOESM1_ESM.zip › Original Image/░⌐┼╘╫Θ╓»20▒╢.tif]
